# The effect of increased atmospheric temperature and CO_2_ concentration during crop growth on the chemical composition and *in vitro* rumen fermentation characteristics of wheat straw

**DOI:** 10.1186/s40104-015-0045-9

**Published:** 2015-11-04

**Authors:** Xiangyu He, Yanping Wu, Min Cai, Chunlong Mu, Weihong Luo, Yanfen Cheng, Weiyun Zhu

**Affiliations:** Jiangsu Key Laboratory of Gastrointestinal Nutrition and Animal Health, Laboratory of Gastrointestinal Microbiology, Nanjing Agricultural University, Nanjing, 210095 China; College of Agriculture, Nanjing Agricultural University, Nanjing, 210095 China

**Keywords:** Chemical composition, CO_2_, *In vitro* digestibility, Temperature, Wheat straw

## Abstract

This experiment was conducted to investigate the effects of increased atmospheric temperature and CO_2_ concentration during crop growth on the chemical composition and *in vitro* rumen fermentation characteristics of wheat straw. The field experiment was carried out from November 2012 to June 2013 at Changshu (31°32′93″N, 120°41′88″E) agro-ecological experimental station. A total of three treatments were set. The concentration of CO_2_ was increased to 500 μmol/mol in the first treatment (CO_2_ group). The temperature was increased by 2 °C in the second treatment (TEM group) and the concentration of CO_2_ and temperature were both increased in the third treatment (CO_2_ + TEM group). The mean temperature and concentration of CO_2_ in control group were 10.5 °C and 413 μmol/mol. At harvesting, the wheat straws were collected and analyzed for chemical composition and *in vitro* digestibility. Results showed that dry matter was significantly increased in all three treatments. Ether extracts and neutral detergent fiber were significantly increased in TEM and CO_2_ + TEM groups. Crude protein was significantly decreased in CO_2_ + TEM group. *In vitro* digestibility analysis of wheat straw revealed that gas production was significantly decreased in CO_2_ and CO_2_ + TEM groups. Methane production was significantly decreased in TEM and CO_2_ + TEM groups. Ammonia nitrogen and microbial crude protein were significantly decreased in all three treatments. Total volatile fatty acids were significantly decreased in CO_2_ and CO_2_ + TEM groups. In conclusion, the chemical composition of the wheat straw was affected by temperature and CO_2_ and the *in vitro* digestibility of wheat straw was reduced, especially in the combined treatment of temperature and CO_2_.

## Introduction

Straw is widely used in China for ruminant production and serves as a potential nutrition source. It undergoes digestion by ruminal microbes [1]. Temperature and CO_2_ levels are important factors regulating crop growth. The atmospheric temperature and concentrations of CO_2_ have increased in the past decades [2]. CO_2_ concentrations have increased by about 40 % since pre-industrial times and reached 391 μmol/mol in 2011 [2]. The increases in temperature and CO_2_ concentrations have had a significant impact on crop production and its biological variables, such as the lengths of crop growth periods and the crop cycle [3]. In general, higher CO_2_ concentrations increase plant production due to higher rates of photosynthesis and water utilization and reduces grain protein concentration, which results in lower grain quality [4]. Meanwhile, the duration of a plant’s developmental stages is extremely sensitive to climate conditions, especially temperature [5]. Experimental warming was shown to shorten phenological stages in wheat, resulting in a 9 % yield decrease per 1 °C increase in temperature [6]. However, limited data are available about the effect of warming and enriched CO_2_ on the nutrient components of wheat straw.

*In vitro* technique has been used as a measure of ruminal degradation of feeds [7, 8] and as an indicator of the digestible dry matter intake (DMI) and growth rate for cattle that are fed cereal straws [9, 10]. This technique primarily measures digestion of soluble and insoluble carbohydrates [7], the amount of gas produced from a feed on incubation, and production of volatile fatty acids, which are a major source of energy for ruminants. This technique also has the potential to reveal associative effects between feeds [10–12]. Therefore, the *in vitro* fermentation technique was employed to evaluate the fermentation of straw by ruminal microbes. Due to the different abilities of ruminal microbiota to utilize nutrients [13, 14], the potential difference in nutrient composition in straw may affect microbial fermentation. Thus, this research supported that the potential difference in nutrient component caused by temperature and CO_2_ variation may also change the utilization of straw by ruminal microbiota.

We hypothesized that an increase in temperature and CO_2_ may affect the nutrient composition of straw and its utilization by ruminal microbiota. The objective of this study was to investigate the effects of increased atmospheric temperature and CO_2_ concentration during crop growth on the chemical composition and *in vitro* fermentation characteristics of wheat straw, which will help to improve the utilization and functionality of wheat straw for ruminants.

## Materials and methods

### Material substrates

The field experiment was carried out at Changshu (31°32′93″N, 120°41′88″E) agro-ecological experimental station, Changshu, Jiangsu Province, China, in a humid, semi-tropical climate. The mean annual temperature is 15.4 °C with rainfall of about 1,054 mm and solar radiation of 4,540 MJ/m^2^/y. The mean annual concentration of CO_2_ is 413 μmol/mol. The soil contained 14.2 g/kg organic matter, 1.1 mg/kg available N, 41.9 mg/kg Olsen-P and 93.3 mg/kg K_2_O.

**Fig. 1:**
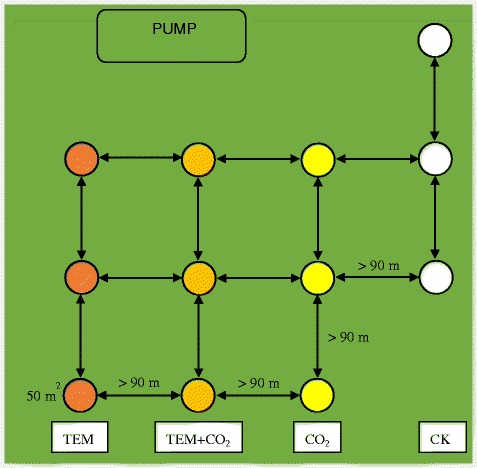
Diagram of the experimental design (CK, control, concentration of CO_2_ 413 μmol/mol and average temperature 10.5 °C; CO_2_, elevated CO_2_ group, concentration of CO_2_ 500 μmol/mol; TEM, elevated temperature group, average temperature 12.5 °C; CO_2_ + TEM, elevated CO_2_ and temperature group, i.e., concentration of CO_2_ 500 μmol/mol, temperature 12.5 °C)

The high-yield winter wheat (*Triticum aestivum L*.) cultivar, Yangmai 14, was sown on November 23, 2012 and harvested on June 13, 2013. A dose of 120 kg N per ha, 60 kg P_2_O_5_ per ha and 120 kg K_2_O per ha were applied as basal fertilizer before sowing and another 120 kg N/ha was top-dressed at jointing following the local wheat management practices. A total of three treatment groups and one control group were set. Each group had three replicates with a randomized complete block design and the area of each replicate plot was 50 m^2^. In order to avoid the cross-contamination, the distances between plots were more than 90 m (Fig. 1). The first treatment (CO_2_ group) was to increase the concentration of CO_2_ to 500 μmol/mol by pumping pure CO_2_ into the ring plot via perforated pipes surrounding the ring from a commercial liquid supplier. The second treatment (TEM group) was to increase the temperature by 2 °C with an infrared heater over the crop canopy. The third treatment (CO_2_ + TEM group) was to increase both CO_2_ concentration and temperature as described in the first two treatments. Li-COR CO_2_ sensors and thermometer were both equipped over canopy and around the ring to automatically control the CO_2_ pumping and canopy air heating, respectively. The control group (CK) was surrounded by the same ring plot without any treatment, with the mean temperature of 10.5 °C and 413 μmol/mol of CO_2_.

At harvesting, the wheat crop was cut above 40–50 cm and the grains were removed with wheat harvester (Lovol, GF60 (4LZ-6 F), China). The whole wheat straw with desiccated leaves were collected and dried in a forced-air oven (Yiheng, PH070, China) for 24 h at 60 °C and then grounded and filtered by a 0.85 mm mesh sieve screen before use.

### Chemical analyses

Samples were analyzed for dry matter (DM, method 930.15), ash (method 942.05), ether extract (EE, method 920.39), crude protein (CP, method 984.13) according to AOAC (1990) [15]. CP was calculated as Kjeldahl N*6.25. Neutral detergent fiber (NDF), acid detergent fiber (ADF) and acid detergent lignin (ADL) were determined by Van Soest et al.’s method [16]. Both ADF and NDF were expressed exclusive of residual ash. Lignin was determined by the solubilization of cellulose with sulfuric acid on the ADF residue [17]. NDF was analyzed without the addition of sodium sulfite and heat stable α-amylase. Hemicellulose was calculated as the difference between NDF and ADF.

### Rumen inocula and sample collection

Four rumen fistulated local goats were used for donor animals. The diet was formulated to meet the maintenance requirement, which included 700 g/kg forage (Chinese wildrye) and 300 g/kg concentrate mixtures (200 g/kg ground corn, 70 g/kg soybean meal, 15 g/kg calcium hydrogen phosphate, 5 g/kg stone powder, 5 g/kg sodium chloride and 5 g/kg mineral and vitamin premix). Rumen fluids were collected into pre-warmed insulated flasks at 2 h after the morning feeding and immediately transported to the laboratory.

All laboratory operations were performed under anaerobic conditions by continuous flushing with CO_2_. The amount of 10 ml rumen fluid, strained through four layers of cheesecloth under anaerobic conditions [18], was inoculated into pre-warmed bottles containing 50 ml medium [19] and 1 g wheat straw as a substrate. The medium was an anaerobic buffer/mineral solution containing per litre: NH_4_HCO_3_ 0.8 g, NaHCO_3_ 7 g, Na_2_HPO_4_ 1.14 g, KH_2_PO_4_ 1.24 g, MgSO_4_ · 7H_2_O 0.12 g, CaCl_2_ · 2H_2_O 0.0132 g, MnCl_2_ · 4H_2_O 0.01 g, CoCl_2_ · 6H_2_O 0.001 g, FeCl_3_ · 6H_2_O 0.008 g, L-Cysteine HCl 0.625 g, NaOH 0.16 g and resazurine 0.001 g. Cultures were incubated at 39 °C for 48 h without agitation. Each sample was analyzed in triplicate as analytic replicates. Gas production and methane emission were measured at 2, 4, 6, 10, 12, 18, 24, 36 and 48 h using the pressure transducer technique [19]. Following readings, the head-space gas was vented, returning the pressure back to ambient conditions. Methane was measured by injecting 30 μL of the head-space gas into gas chromatograph (Shimadzu, GC-14B, Japan) equipped with thermal conductivity detector. The temperatures of the column, injector and flame ionization detector were 80, 100 and 120 °C, respectively. 99.99 % methane was used as external standard. At the end of 24 and 48 h, pH was measured immediately upon removing the crimp seals and stoppers from the bottles, and approximately 10 mL of supernatant was collected for the analysis of VFAs, ammonia nitrogen (NH_3_-N), and microbial crude protein (MCP). The substrate residues were collected for the determination of DM, NDF, and ADF, and the corresponding dry matter digestibility (DMD), neutral detergent fiber digestibility (NDFD), and acid detergent fiber digestibility (ADFD) were calculated.

### Determination of pH and fermentation end products

Culture pH was measured by a pH meter (Schott, Germany). The VFAs were determined by gas chromatography (Shimadzu, GC-14B, Japan) according to Mao et al. [20]. The NH_3_-N concentration was measured by the indophenol method [21]. The MCP concentration was determined according to Makkar et al. [22] by spectrophotometer (Thermo Fisher Scientific, NanoDrop2000c, Wilmington, DE, USA).

### Statistical analysis

The data were tabulated as mean ± standard error for all parameters and analyzed using the one-way analysis of variance (ANOVA) procedure with SPSS 17.0 (SPSS, Chicago, IL, USA). The differences of the means of the treatments were tested by using Duncan’s new multiple range test. The correlations between the different parameters were established by calculating Pearson’s correlation coefficient. In all the analyses, significant effects were declared at *P* < 0.05.

## Results

### Chemical composition

The effects of temperature and CO_2_ on the chemical composition of the wheat straw were shown in Table 1. ADF, hemicellulose, and acid detergent lignin were not affected in all treatments (*P* > 0.05). In CO_2_ group, DM increased significantly (*P* < 0.05). In TEM group, ash decreased significantly (*P* < 0.05) and EE increased significantly (*P* < 0.05). In CO_2_ + TEM group, DM and NDF significantly (*P* < 0.05) increased, while ash and CP significantly decreased (*P* < 0.05). Among the three treatments, the NDF content in CO_2_ + TEM was significantly higher than that in TEM and CO_2_ groups (*P* < 0.05). CP and EE in CO_2_ + TEM and CO_2_ were significantly lower than that in TEM.

**Table 1:** Composition of wheat straw under different treatments (dry matter basis)

Item, g/100 g	CK	CO_2_	TEM	CO_2_ + TEM	S.E.M	*P*- Value^1^
Dry matter	94.37 ± 0.11^a^	95.25 ± 0.31^bc^	94.60 ± 0.12^ab^	95.57 ± 0.24^c^	0.17	*
Crude ash	4.46 ± 0.10^a^	4.56 ± 0.14^a^	3.49 ± 0.13^b^	3.69 ± 0.13^b^	0.15	*
Crude protein	1.45 ± 0.05^ab^	1.29 ± 0.03^bc^	1.59 ± 0.06^a^	1.17 ± 0.06^c^	0.05	*
Ether extracts	0.70 ± 0.07^b^	0.57 ± 0.04^b^	0.92 ± 0.02^a^	0.64 ± 0.02^b^	0.04	*
Neutral detergent fiber	82.95 ± 0.03^a^	82.98 ± 0.19^a^	82.96 ± 0.23^a^	84.72 ± 0.16^b^	0.24	*
Acid detergent fiber	52.47 ± 0.76	52.52 ± 0.66	52.59 ± 0.46	53.96 ± 0.69	0.33	ns
Hemicellulose	30.48 ± 0.77	30.46 ± 0.84	30.65 ± 0.37	31.09 ± 0.28	0.27	ns
Acid detergent lignin	6.31 ± 0.24	6.45 ± 0.15	6.57 ± 0.13	7.02 ± 0.29	0.12	ns

*CK* control, concentration of CO_2_ 413 μmol/mol and average temperature 10.5 °C; *CO*_*2*_ elevated CO_2_ group, concentration of CO_2_ 500 μmol/mol; *TEM* elevated temperature group, average temperature 12.5 °C; *CO*_*2*_ + *TEM*, elevated CO_2_ and temperature group, i.e., concentration of CO_2_ 500 μmol/mol, temperature 12.5 °C. The same as below

^a,b,c^Values within a column with different superscript letters are significantly different (*P* < 0.05)

^1^ns = non-significant (*p* > 0.05), * = significant (*P* < 0.05)

### Apparent *in vitro* DM, NDF, and ADF digestibility

The apparent *in vitro* DM, NDF, and ADF digestibility of the wheat straw were presented in Table 2. DMD, NDFD, and ADFD were not affected by CO_2_ and TEM treatments (*P* > 0.05). But DMD and NDFD were significantly (*P* < 0.05) decreased at 24 h in CO_2_ + TEM group, and no significant difference was observed at 48 h.

**Table 2:** Effects of CO_2_ and temperature treatments on DM, NDF and ADF digestibility at 24 and 48 h of wheat straw

Index^1^, g/100 g	Time, h	CK	CO_2_	TEM	CO_2_ + TEM	S.E.M	*P*- Value^2^
DMD	24	16.41 ± 0.44^a^	14.88 ± 0.47^ab^	15.73 ± 0.60^ab^	14.60 ± 0.56^b^	0.31	ns
	48	26.26 ± 0.63	25.43 ± 0.69	26.15 ± 0.55	24.32 ± 0.52	0.35	ns
NDFD	24	17.72 ± 0.49^a^	16.67 ± 0.32^ab^	17.02 ± 0.29^ab^	16.45 ± 0.41^b^	0.22	ns
	48	32.59 ± 0.79	31.39 ± 0.56	31.55 ± 0.70	30.49 ± 1.12	0.41	ns
ADFD	24	12.24 ± 0.91	11.03 ± 0.55	10.82 ± 0.50	10.39 ± 0.44	0.34	ns
	48	28.69 ± 1.20	27.36 ± 0.66	27.50 ± 0.74	26.35 ± 0.40	0.42	ns

^1^ *DMD* dry matter digestibility; *NDFD* Neutral detergent fiber digestibility; *ADFD* Acid detergent fiber digestibility

^2^ns = non-significant (*P* > 0.05)

^a,b^Values within a column with different superscript letters are significantly different (*P* < 0.05)

### *In vitro* gas and methane production

As shown in Table 3, the maximum gas production of 76.38 ± 1.28 mL was caused by CK during 48 h of incubation, which was closely followed by TEM (73.63 ± 2.24 mL) and CO_2_ (68.16 ± 1.55 mL), while CO_2_ + TEM caused a minimum of 66.44 ± 1.49 mL. GP was significantly decreased in CO_2_ and CO_2_ + TEM groups at 48 h (*P* < 0.05). Among the three treatments, gas production in CO_2_ + TEM group was significantly lower than that in TEM (*P* < 0.05). During 48 h of incubation, the maximum methane production was caused by CO_2_ (27.58 ± 1.38 mL), followed by CK (25.80 ± 1.15 mL), CO_2_ + TEM (20.67 ± 0.21 mL), and TEM (17.88 ± 0.77 mL). At 24 h, MP was significantly decreased in TEM group (*P* < 0.05). Among the three treatments, MP in CO_2_ and CO_2_ + TEM groups was significantly higher than that in TEM (*P* < 0.05). At 48 h, MP was significantly decreased in TEM and CO_2_ + TEM groups (*P* < 0.05). Among the three treatments, MP in CO_2_ group was significantly higher than that in TEM and CO_2_ + TEM groups (*P* < 0.05).

**Table 3:** Effects of CO_2_ and temperature treatments on *in vitro* pH, concentration of ammonia nitrogen (mg/100 ml) and microbial crude (mg/100 mL) protein, gas and methane production at 24 and 48 h of wheat straw

Index^1^, g/100 g	Time, h	CK	CO_2_	TEM	CO_2_ + TEM	S.E.M	*P*- Value^2^
pH	24	6.62 ± 0.01^a^	6.67 ± 0.01^b^	6.64 ± 0.01^a^	6.69 ± 0.01^b^	0.01	*
	48	6.56 ± 0.01^a^	6.58 ± 0.01^ab^	6.56 ± 0.01^a^	6.61 ± 0.01^b^	0.01	*
NH_3_-N	24	3.34 ± 0.03^a^	3.22 ± 0.03^b^	3.24 ± 0.02^b^	3.24 ± 0.03^b^	0.30	*
(mg/100 mL)	48	5.17 ± 0.03^a^	4.60 ± 0.08^b^	4.71 ± 0.06^b^	4.55 ± 0.07^b^	0.08	*
MCP	24	8.29 ± 0.14^a^	7.13 ± 0.21^b^	8.31 ± 0.13^a^	7.09 ± 0.43^b^	0.21	*
(mg/100 mL)	48	10.46 ± 0.19^a^	7.93 ± 0.19^b^	8.63 ± 0.32^b^	7.72 ± 0.55^b^	0.36	*
Gas production (mL)	24	25.41 ± 0.71	25.78 ± 0.46	25.80 ± 1.25	23.51 ± 1.21	0.50	ns
	48	76.38 ± 1.28^a^	68.16 ± 1.55^bc^	73.63 ± 2.24^ab^	66.44 ± 1.49^c^	1.41	*
Methane production (mL)	24	6.41 ± 0.10^a^	7.84 ± 0.59^a^	3.95 ± 0.58^b^	7.25 ± 0.69^a^	0.50	*
	48	25.80 ± 1.15^a^	27.58 ± 1.38^a^	17.88 ± 0.77^b^	20.67 ± 0.21^b^	1.24	*

^a,b^ Values within a column with different superscript letters are significantly different (*P* < 0.05)

^1^VFA: volatile fatty acid. ^2^ns = non-significant (*P* > 0.05)

### pH, NH_3_-N, MCP, and VFA

The pH varied in different groups, but was still in the normal range throughout the incubation, ranging from 6.56 to 6.69 (Table 3). The pH was significantly increased in CO_2_ group at 24 h and in CO_2_ + TEM group at 24 and 48 h (*P* < 0.05). Among the three treatments, the pH in TEM group was significantly lower than that in CO_2_ group at 24 h and CO_2_ + TEM group at 24 and 48 h (*P* < 0.05). NH_3_-N was significantly decreased in all three treatments at 24 and 48 h (*P* < 0.05) and no significant difference was observed among the treatments. MCP was significantly decreased in all treatments at 48 h (*P* < 0.05) and in CO_2_ and CO_2_ + TEM groups at 24 h (*P* < 0.05). Among the three treatments, MCP in TEM group was significantly higher than that in CO_2_ and CO_2_ + TEM at 24 h (*P* < 0.05) and no significant difference was observed at 48 h.

The effects of temperature and CO_2_ on the concentration of VFA were shown in Table 4. The concentrations of propionate, butyrate, and the acetate: propionate ration were not affected in all treatments (*P* > 0.05), while the concentration of TVFA was significantly decreased by the CO_2_ + TEM treatment (*P* < 0.05). Meanwhile, the concentration of TVFA was significantly decreased by CO_2_ at 24 h (*P* < 0.05). Among the three treatments, the concentration of TVFA in CO_2_ + TEM was significantly lower than that in CO_2_ and TEM at 24 h (*P* < 0.05) and significantly lower than that in TEM at 48 h (*P* < 0.05). The concentration of acetate was significantly decreased in CO_2_ and CO_2_ + TEM groups at 24 and 48 h (*P* < 0.05). Among the three treatments, the concentration of acetate in TEM was significantly higher than that in CO_2_ and CO_2_ + TEM at 24 h (*P* < 0.05), and it was significantly higher than that in CO_2_ + TEM at 48 h.

**Table 4:** Effects of CO_2_ and temperature treatments on *in vitro* VFA at 24 and 48 h of wheat straw

Index, mmol/L	Time, h	CK	CO_2_	TEM	CO_2_ + TEM	S.E.M	*P*- Value^2^
TVFA^1^	24	17.52 ± 0.17^a^	15.04 ± 0.02^b^	16.90 ± 0.38^a^	12.42 ± 0.23^c^	0.63	*
	48	38.83 ± 1.96^a^	34.95 ± 0.30^ab^	37.11 ± 1.41^a^	32.50 ± 1.07^b^	0.91	*
Acetate	24	11.10 ± 0.29^a^	9.18 ± 0.23^b^	10.32 ± 0.30^a^	6.95 ± 0.07^c^	0.52	*
	48	23.97 ± 0.94^a^	21.17 ± 0.45^bc^	22.23 ± 0.73^ab^	19.17 ± 0.63^c^	0.61	*
Propionate	24	4.94 ± 0.14	4.54 ± 0.26	5.12 ± 0.47	4.19 ± 0.32	0.17	ns
	48	11.37 ± 0.61	10.65 ± 0.19	11.42 ± 0.53	10.24 ± 0.41	0.25	ns
Butyrate	24	1.33 ± 0.12	1.14 ± 0.03	1.25 ± 0.11	1.10 ± 0.08	0.07	ns
	48	2.98 ± 0.37	2.49 ± 0.07	2.81 ± 0.20	2.43 ± 0.11	0.12	ns
Acetate : propionate	24	2.25 ± 0.10	2.04 ± 0.18	2.06 ± 0.25	1.68 ± 0.13	0.1	ns
	48	2.11 ± 0.04	1.99 ± 0.06	1.95 ± 0.06	1.88 ± 0.05	0.03	ns

^a,b,c^ Values within a column with different superscript letters are significantly different (*P* < 0.05)

^1^VFA: volatile fatty acid. ^2^ns = non-significant (*P* > 0.05), * = significant (*P* < 0.05)

### The correlation between chemical constituents and fermentation products of wheat straw after 48 h fermentation

The relationships between the chemical constituents and fermentation products of the wheat straw were shown in Table 5. DM was negatively correlated (*P* < 0.05) with NH_3_-N, GP, acetate, and the TVFA concentration. NDF was negatively correlated (*P* < 0.05) with GP, TVFA, and acetate. ADL was negatively correlated (*P* < 0.05) with NDFD and GP. However, ash was positively correlated (*P* < 0.05) with the MCP concentration. CP was positively correlated (*P* < 0.05) with GP, MP, and acetate. Negative correlation (*P* < 0.05) was also observed between EE and MP.

**Table 5:** Correlation between chemical composition and *in vitro* fermentation characteristics of wheat straw

Item^1^	DMD	NDFD	ADFD	NH_3_-N	MCP	GP	MP	Acetate	Propionate	Butyrate	TVFA
DM	−0.49	−0.35	−0.39	−0.60	−0.47	−0.60	−0.05	−0.71	−0.43	−0.40	−0.62
*P*-Value^2^	ns	ns	ns	*	ns	*	ns	*	ns	ns	*
Ash	0.12	0.34	0.31	0.43	0.66	0.21	−0.84	0.41	0.13	0.21	0.33
*P*-Value	ns	ns	ns	ns	*	ns	*	ns	ns	ns	ns
EE	0.39	0.07	−0.15	−0.08	−0.15	0.40	−0.60	0.23	0.24	0.07	0.23
*P*-Value	ns	ns	ns	ns	ns	ns	*	ns	ns	ns	ns
CP	0.49	0.31	0.31	0.48	0.29	0.47	−0.25	0.60	0.46	0.38	0.57
*P*-Value	ns	ns	ns	ns	ns	*	ns	*	ns	ns	ns
NDF	−0.52	−0.43	−0.52	−0.53	−0.50	−0.67	−0.30	−0.73	−0.49	−0.44	−0.67
*P*-Value	ns	ns	ns	ns	ns	*	ns	*	ns	ns	*
ADF	0.13	−0.45	−0.42	−0.24	−0.27	−0.47	−0.38	−0.35	−0.32	−0.10	−0.33
*P*-Value	ns	ns	ns	ns	ns	ns	ns	ns	ns	ns	ns
HC	−0.56	0.05	−0.05	−0.25	−0.20	−0.19	−0.04	−0.39	−0.26	−0.43	−0.38
*P*-Value	ns	ns	ns	ns	ns	ns	ns	ns	ns	ns	ns
ADL	−0.13	−0.67	−0.32	−0.42	−0.35	−0.69	−0.42	−0.46	−0.46	−0.31	−0.54
*P*-Value	ns	*	ns	ns	ns	*	ns	ns	ns	ns	ns

^1^ *DM* dry matter; *CP* crude protein; *EE* ether extracts; *NDF*neutral detergent fiber; *ADF* acid detergent fiber; *HC* hemicellulose; *ADL* acid detergent lignin; *DMD* dry digestibility; *NDFD* NDF digestibility; *ADFD* ADF digestibility; *GP* gas production; *MP* methane production; *VFA* volatile fatty acid; *NH*_*3*_-*N* ammonia nitrogen; *MCP* microbial crude protein

^2^ns = non-significant (*P* > 0.05), * = significant (*P* < 0.05)

## Discussion

In the present study, elevated temperature and CO_2_ levels were found to increase the DM, EE and NDF contents and reduce the ash and CP contents. Rosenzweig et al. [23] reported that increasing the CO_2_ concentration and temperature might lead to the increase of photosynthesis, carbohydrate fixation, respiration and transpiration in the plants. Increased photosynthesis and carbohydrate fixation might result in the increase of growth rate and plant biomass [24], which would lead to the increase of dry matter in the plants. Meanwhile, the increased respiration and transpiration would lead to the increase of total leaf area, which could increase the evaporation of water, decrease the water content, and thus relatively increase the dry matter content in plants [25]. Carten et al. [26] reported that a combined increase in temperature and CO_2_ concentration could increase the biomass of three genotypes of *Lotus corniculatus*. Hocking et al. [27] also showed that shoot to root dry matter rations of both wheat and maize increased with CO_2_ enrichment. The allocation of nutrients in the plants was also affected by increasing temperature and CO_2_ concentration. Asseng et al. [28] reported that temperature and CO_2_ increased the protein concentration of grain and affected the composition of amino acids [29]. As a result, the protein concentration in straw would decrease and the NDF content would increase. Erice et al. [30] reported that elevated temperature and CO_2_ significantly reduced the CP content of alfalfa and had the potential to enhance its NDF and ADF contents. Wayne et al. [31] also reported that elevated temperature and CO_2_ reduced the CP content of leaves in yellow birch. Little is known about the effects of elevated temperature on the content and distribution of EE in plants, it may be due to the increase of photosynthesis [32].

By using *in vitro* fermentation, we found the digestibility and degradation of wheat straw decreased with the increase of temperature and CO_2_. The combined increase of temperature and CO_2_ had the most significant impacts, which was revealed by the decrease of DMD, NDFD, GP, MP and concentrations of NH_3_, MCP, TVFA and acetate. Increase of CO_2_ decreased GP and concentrations of NH_3_, MCP, TVFA and acetate. Increase of temperature only decreased MP and the concentrations of NH_3_ and MCP. The decrease of DMD and NDFD might be due to the decrease of CP and increase of DM and NDF in the CO_2_ + TEM group. Previous research demonstrated that a high NDF level directly decreased *in vitro* DM and NDF disappearance of several ruminant feeds [33]. Iantcheva et al. [34] demonstrated a highly negative effect of NDF on the digestibility of grass hay. Elghandour et al. [35] also showed that the digestibility of four fibrous feeds decreased under increased DM and NDF levels.

We found that the combined increase of temperature and CO_2_ significantly decreased the concentration of MCP during *in vitro* fermentation, which suggested that the numbers of microorganisms in the fermentation decreased. The decrease of MCP might be related to the decrease of NH_3_-N and microbial attachment to the plant. Previous studies showed that a decreased concentration of NH_3_-N might result in the decrease of MCP as NH_3_-N could be used for microbial protein synthesis [36]. In addition, increased NDF might also lead to the decrease of microbial attachment to the plants [37].

The increase of NDF led to the decrease of soluble carbohydrate fractions in wheat straw, resulting in the decrease of end products of microbial fermentation [38]. Gases (mainly CO_2_ and methane) are important end products of the fermentation [39]. Sallam et al. [40] reported a negative correlation between cell wall fraction and *in vitro* gas production. Getachew et al. [33] also observed reduced gas production by increasing NDF content in the fermentation of several ruminant feeds. The negative relationship between NDF level and gas production was also reported in the fermentation of tropical trees by Nsahlai et al. [41] and Larbi et al. [42]. Methane was mainly produced via methanogenesis by methanogens such as *Methanobrevibacter smithii* [43]. Besides the decreased digestibility of wheat straw, the increase of EE might be another reason for the decrease of methane production. Johnson et al. [44] reported that increased lipid content in the feed was thought to decrease methanogenesis through inhibition of protozoa, increase of propionic acid, and biohydrogenation of unsaturated fatty acids. Dohme et al. [45] also reported that lipid could inhibit the growth of methanogens directly through binding to the cell membrane and interrupting membrane transport. Lee et al. [46] observed a negative relationship between methane production and EE in the fermentation of feed ingredients. A negative correlation between methane production and EE content was also observed in the present study, which implied that the EE might be a potential factor regulating methane production from the rumen.

The most important water soluble end products of the fermentation are NH_3_-N and VFA representing the metabolism of nitrogen and carbon [47]. The decrease of NH_3_-N was due to the decrease of the CP content of the wheat straw. Chen et al. [48] suggested that NH_3_-N concentration was significantly affected by changing the levels of CP in cattle feed. Ghorbani et al. [49] reported that increased CP could increase the NH_3_-N concentration. The production of VFA represented the main supply of metabolizable energy for ruminants [50]. Nsahlai et al. [41] and Larbi et al. [42] reported that a negative relationship between NDF level and VFA was observed in the fermentation of 23 browses of the genus *Sesbania* and fodder trees. In the present study, the decrease of VFA was also observed and it was mainly due to the decrease of acetate, as no significant difference was observed in the production of propionate and butyrate. As summarized by Bergman [51], acetate was the major end product of microbial fermentation in the rumen and it contributed about 40 % of a ruminant’s daily energy requirements. Therefore, the decrease in acetate production may indicate a reduction of energy supply for ruminants.

## Conclusions

Elevated temperature and concentration of CO_2_ changed the chemical composition of wheat straw in an adverse fashion, which resulted in the decrease of digestibility and degradation of wheat straw. Most importantly, the decrease of the concentration of volatile fatty acids, especially acetate, may reduce the energy source for ruminants, thus decreasing the performance of livestock.
